# The role of the plasmid-mediated fluoroquinolone resistance genes as resistance mechanisms in pediatric infections due to Enterobacterales

**DOI:** 10.3389/fcimb.2023.1249505

**Published:** 2023-10-09

**Authors:** Latania K. Logan, Laura Rojas Coy, Claire E. Pitstick, Steven H. Marshall, Rachel L. Medernach, T. Nicholas Domitrovic, Sreenivas Konda, Nadia K. Qureshi, Andrea M. Hujer, Xiaotian Zheng, Susan D. Rudin, Robert A. Weinstein, Robert A. Bonomo

**Affiliations:** ^1^ Pediatrics, Emory University School of Medicine, Atlanta, GA, United States; ^2^ Department of Pediatrics, Children’s Healthcare of Atlanta, Atlanta, GA, United States; ^3^ Pediatrics, Rush University Medical Center, Chicago, IL, United States; ^4^ Research Service, Louis Stokes Cleveland Department of Veterans Affairs Medical Center, Cleveland, OH, United States; ^5^ Molecular Biology, and Microbiology, Case Western Reserve University School of Medicine, Cleveland, OH, United States; ^6^ Medicine, Rush University Medical Center, Chicago, IL, United States; ^7^ Biostatistics, University of Illinois at Chicago, Chicago, IL, United States; ^8^ Pediatrics, Loyola University Medical Center, Maywood, IL, United States; ^9^ Medicine, Case Western Reserve University School of Medicine, Cleveland, OH, United States; ^10^ Microbiology, Ann & Robert H. Lurie Children’s Hospital of Chicago, Chicago, IL, United States; ^11^ Pathology, Northwestern Feinberg School of Medicine, Chicago, IL, United States; ^12^ Department of Medicine, Cook County Health, Chicago, IL, United States; ^13^ Biochemistry, Pharmacology, Proteomics and Bioinformatics, Case Western Reserve University School of Medicine, Cleveland, OH, United States; ^14^ Case Western Reserve University (CWRU)-Cleveland VA Medical Center (VAMC) Center for Antimicrobial Resistance and Epidemiology (Case VA CARES), Cleveland, OH, United States

**Keywords:** epidemiology, gram-negative bacteria, Enterobacterales infections, fluoroquinolone resistance, beta-lactamases, children, antibiotic resistance, *mcr*-9

## Abstract

**Introduction:**

Fluoroquinolones (FQs) are not commonly prescribed in children, yet the increasing incidence of multidrug-resistant (MDR) Enterobacterales (Ent) infections in this population often reveals FQ resistance. We sought to define the role of FQ resistance in the epidemiology of MDR Ent in children, with an overall goal to devise treatment and prevention strategies.

**Methods:**

A case–control study of children (0–18 years) at three Chicago hospitals was performed. Cases had infections by FQ-susceptible, β-lactamase-producing (*bla*) Ent harboring a non- or low-level expression of PMFQR genes (PMFQS Ent). Controls had FQR infections due to *bla* Ent with expressed PMFQR genes (PMFQR Ent). We sought *bla* genes by PCR or DNA (BD Max Check-Points assay^®^) and PMFQR genes by PCR. We performed rep-PCR, MLST, and *E. coli* phylogenetic grouping. Whole genome sequencing was additionally performed on PMFQS Ent positive isolates. Demographics, comorbidities, and device, antibiotic, and healthcare exposures were evaluated. Predictors of infection were assessed.

**Results:**

Of 170 β-lactamase-producing Ent isolates, 85 (50%) were FQS; 23 (27%) had PMFQR genes (PMFQS cases). Eighty-five (50%) were FQR; 53 (62%) had PMFQR genes (PMFQR controls). The median age for children with PMFQS Ent and PMFQR Ent was 4.3 and 6.2 years, respectively (*p* = NS). Of 23 PMFQS Ent, 56% were *Klebsiella* spp., and of 53 PMFQR Ent, 76% were *E. coli*. The most common *bla* and PMFQR genes detected in PMFQS Ent were *bla*_SHV ESBL_ (44%) and *oqxAB* (57%), and the corresponding genes detected in PMFQR Ent were *bla*_CTX-M-1-group ESBL_ (79%) and *aac(6’)-Ib-cr* (83%). Whole genome sequencing of PMFQS Ent revealed the additional presence of *mcr-9*, a transferable polymyxin resistance gene, in 47% of isolates, along with multiple plasmids and mobile genetic elements propagating drug resistance. Multivariable regression analysis showed that children with PMFQS Ent infections were more likely to have hospital onset infection (OR 5.7, 95% CI 1.6–22) and isolates containing multiple *bla* genes (OR 3.8, 95% CI 1.1–14.5). The presence of invasive devices mediated the effects of healthcare setting in the final model. Differences in demographics, comorbidities, or antibiotic use were not found.

**Conclusions:**

Paradoxically, PMFQS Ent infections were often hospital onset and PMFQR Ent infections were community onset. PMFQS Ent commonly co-harbored multiple *bla* and PMFQR genes, and additional silent, yet transferrable antibiotic resistance genes such as *mcr-9*, affecting therapeutic options and suggesting the need to address infection prevention strategies to control spread. Control of PMFQS Ent infections will require validating community and healthcare-based sources and risk factors associated with acquisition.

## Introduction

Multidrug-resistant (MDR) Enterobacterales (Ent) infections are associated with significant morbidity and mortality and are a growing concern among children and healthy populations in the US (Logan et al., 2014; Logan et al., 2015b). This dramatic rise in resistance is globally attributed mainly to extended-spectrum β-lactamase (ESBL)-producing Enterobacterales and carbapenem-resistant Enterobacterales (CRE) (Logan et al., 2015a; Lukac et al., 2015; Logan and Weinstein, 2017). However, increasing resistance to several other classes of antibiotics is co-occurring, including to the fluoroquinolones (FQs), a class of antibiotics that are not commonly prescribed in children, yet the increasing incidence of MDR Ent infections in the pediatric population often reveals FQ resistance (Medernach and Logan, 2018).

Among MDR Enterobacterales, *Escherichia coli* harboring *bla*_CTX-M_ are the most common ESBL-producing Enterobacterales circulating, and the bacteria often possess resistance genes to other important antibiotic classes including fluoroquinolones, aminoglycosides, trimethoprim-sulfamethoxazole, and tetracyclines (Cantón and Coque, 2006; Price et al., 2013). In adults, fluoroquinolone resistance (FQR) in Gram-negative bacteria has been linked to both chromosomal and plasmid-mediated resistance mechanisms and most likely associated with the increase in use of these antibiotics during the 1980s (Robicsek et al., 2006; Mathers et al., 2015). However, there has been an increasing prevalence of global fluoroquinolone-resistant clones of *E. coli*, including among healthy populations, despite demonstrated decreases in fluoroquinolone use (Tchesnokova et al., 2018; Pitout et al., 2022).

Phenotypic expression of FQR among Enterobacterales often involves chromosomal mechanisms of resistance, specifically mutations in *gyrA* and *parC* genes of the quinolone resistance determining region (QRDR) in *E. coli, Klebsiella* spp., and *Enterobacter* spp (Chen et al., 2012). Plasmid-mediated mechanisms of FQR often have lower levels of expression, and therefore the presence of these transmissible genetic mechanisms of resistance among Enterobacterales isolates may be underrecognized when chromosomal resistance mechanisms are not present (Diancourt et al., 2005; Chen et al., 2012).

As fluoroquinolones are uncommonly prescribed in children, the reasons for the increases in FQR Enterobacterales among children are unclear. Studies assessing FQR in Enterobacterales among children are limited, including the analysis of plasmid-mediated (PM) resistance mechanisms associated with the FQR phenotype (PMFQR) and the FQS phenotype (PMFQS) (Medernach and Logan, 2018; Logan et al., 2019b). Therefore, we examined this population to determine which children had a higher likelihood of infections with Enterobacterales resistant to β-lactam antibiotics, yet variable susceptibilities to fluoroquinolone antibiotics, and determined the genotypes, host factors, and exposures associated with infection. We seek to define the role of FQR in the epidemiology of MDR Enterobacterales colonization and infection in children, with an overall goal to devise effective treatment and prevention strategies.

## Methods

### Study setting

Isolates were obtained from three academic centers in the metropolitan Chicago area. Hospital A is a 115-bed children’s hospital within a tertiary care academic medical center that has a step-down care unit, a pediatric ward, a mother–newborn infant unit, and cardiac, neonatal, and pediatric intensive care units (CICU, NICU, and PICU). Hospital B is a 125-bed children’s hospital within an academic medical center, and contains general pediatric and newborn infant wards, as well as a NICU and PICU. Hospital C is a freestanding children’s academic medical center with 288 beds that provides complex quaternary services, such as pediatric solid organ and bone marrow transplantation.

### Descriptive study design

#### Study population

This study included patients aged 0 to 18.99 years who had clinical cultures positive for Enterobacterales with extended-spectrum cephalosporin and/or carbapenem resistance and the suspected presence of a β-lactamase gene based on clinical microbiology laboratory testing results. Isolates were grouped and further characterized based on susceptibility to fluoroquinolones. Infections were diagnosed between 1 January 2011 and 31 December 2016, and only the first infection episode per pediatric patient was included. The study was approved by the institutional review boards of the three participating institutions and the need for informed consent was waived by all institutions.

#### Antibiotic susceptibility testing in Enterobacterales

The clinical microbiology laboratories of Hospitals A–C phenotypically analyzed presumed β-lactamase-producing Enterobacterales isolates via the Vitek 2 microbial identification system (bioMérieux, Athens, GA) or by the MicroScan WalkAway system (Siemens Healthcare Diagnostics, Tarrytown, NY). Screening for production of extended-spectrum β-lactamases involved testing with one or more of the following agents: ceftazidime, ceftriaxone, cefotaxime, aztreonam, or cefpodoxime, based on guidance from the Clinical and Laboratory Standards Institute (CLSI) (Clinical and Laboratory Standards Institute, 2023). ESBL production was confirmed on the automated instruments or by disk diffusion assays (BBL; Becton, Dickinson and Company, Sparks, MD) or by the measurement of minimum inhibitory concentrations (MICs) of cefotaxime or ceftazidime in the presence and absence of clavulanic acid. The ESBL phenotype is confirmed based on CLSI recommendations during the time period in which the isolates were analyzed (Clinical and Laboratory Standards Institute, 2023).

Isolates that displayed the carbapenemase phenotype were non-susceptible to all extended-spectrum cephalosporins (cefotaxime, ceftazidime, or ceftriaxone) and resistant to one or more carbapenem (imipenem, meropenem, doripenem, or ertapenem) as per Centers for Disease Control and Prevention (CDC) criteria (Centers for Disease Control and Prevention, 2015). Carbapenemase production was confirmed by phenotypic testing or molecular assays, based on guidance available during the time period in which the isolates were analyzed, as appropriate (Birgy et al., 2012; Centers for Disease Control and Prevention, 2019).

##### Determination of mechanisms of β-lactam resistance

Genomic DNA was purified from Enterobacterales isolates confirmed with an ESBL or carbapenemase-producing phenotype (DNeasy blood and tissue kit, Qiagen, Inc. Valencia, CA). DNA Microarray (Check-MDR CT101 and CT103XL; Check-Points, Wageningen, Netherlands) or polymerase chain reaction (PCR) was utilized for confirmation of the presence of *bla* genes in Enterobacterales isolates as previously described by our laboratory and according to the manufacturer’s protocol (Logan et al., 2016; Powell et al., 2017).

##### Determination of mechanisms of fluoroquinolone resistance

To investigate the presence of FQR determinants among β-lactamase-producing Enterobacterales that were both phenotypically susceptible and resistant to fluoroquinolones, we analyzed the quinolone resistance-determining region (QRDR) located on the bacterial chromosome and assessed for plasmid-mediated mechanisms of resistance using CLSI standards (Clinical and Laboratory Standards Institute, 2023). Briefly, assays for mutations in *gyrA* and *parC* genes of the QRDR (in *E. coli, Klebsiella* spp., and *Enterobacter* spp.) and for plasmid-mediated resistance mechanisms were performed by PCR and/or deoxyribonucleic acid (DNA) sequencing (Robicsek et al., 2006). Extraction of genomic DNA followed by amplification and sequencing were performed using primers and methods as previously described (Cano et al., 2009; Endimiani et al., 2009; Hujer et al., 2009). Specific PMFQR genes for analysis included *aac(6’)-Ib-cr*, *qepA*, *qnrA, qnrB, qnrC, qnrD, qnrS, oqxA*, and *oqxB* and represented transmissible elements previously reported in Enterobacterales (Chen et al., 2012; Jacoby, 2017; Logan et al., 2019b).

##### Nomenclature and characterization of Enterobacterales isolates

*E. coli* was classified into one of four phylogenetic groups (A, B1, B2, and D) using a well-established multiplex PCR-based technique (Bingen-Bidois et al., 2002). Multi-locus sequence typing (MLST) was performed for PMFQR Ent isolates using the Pasteur scheme [Pasteur website (http://www.pasteur.fr/recherche/genopole/PF8/mlst/)] to identify sequence types of ESBL and carbapenemase-producing strains of *E. coli* and *K. pneumoniae*, and the Oxford scheme [PubMLST website (https://pubmlst.org/ecloacae/)] was likewise used on *Enterobacter cloacae* isolates as previously described by our laboratory and others (Diancourt et al., 2005; Jaureguy et al., 2008; Logan et al., 2016; Jolley et al., 2018).

##### Whole genome sequencing on FQS Enterobacterales strains

For PMFQS Ent strains, DNA was extracted using the MasterPure Gram Positive DNA purification kit following the manufacturer’s instructions (Epicentre, Madison, WI). Libraries were prepared using the Oxford Nanopore Technology’s Rapid Barcoding Kit and whole genome sequencing (WGS) was performed in an ONT MinION. The genome was *de novo* assembled with Flye 2.9, polished with Medaka 1.6 annotated using BV-BRC (Bacterial and viral bioinformatics resource center) and confirmed with BLAST (Basic Local Alignment Search Tool, NCBI). Resistome and plasmid types were determined using ResFinder 4.0 and Plasmidfinder 2.1, respectively, from the Center for Genomic Epidemiology (https://www.genomicepidemiology.org/services/).

### Analytic study design

We used a retrospective case–control study design to assess factors associated with infection due to β-lactam-resistant Enterobacterales, which were phenotypically susceptible to fluoroquinolones yet possessed an FQR gene. We selected as cases those who had infections by FQ-susceptible, β-lactam-resistant Enterobacterales harboring a non- or low-level expression of PMFQR genes (PMFQS Ent). We chose to analyze mechanisms of FQR in detail because this antibiotic class is not commonly used in children, yet 50% of the isolates were fluoroquinolone resistant.

Children with β-lactam-resistant Enterobacterales infections that were phenotypically resistant to fluoroquinolones and in which we had detected an FQR gene (PMFQR Ent) were selected as controls. Specifically, the control group included children with infections that were resistant to extended-spectrum cephalosporins, carbapenem, and fluoroquinolone antibiotics to understand differences between children who had Enterobacterales infections that were phenotypically susceptible to fluoroquinolones and those who had fluoroquinolone phenotypically resistant infections.

Only pediatric patients with clinical infections were included, as determined by using standard criteria defined by the CDC National Healthcare Safety Network and by a study investigator case review (Bingen-Bidois et al., 2002).

### Covariates

Multiple variables were analyzed as potential factors associated with PMFQS Ent infection based on known associations for acquisition in adults and children including (1) demographics (age, gender, and race/ethnicity); (2) comorbid conditions (as defined by ICD-9/ICD-10 codes); (3) recent healthcare exposures in both inpatient and outpatient settings, including hospitalization and/or procedures in the previous 30 days; (4) all recent antibiotic exposures in the 40 days prior to culture; and (5) presence, number, and type of invasive medical devices.

### Statistical analysis

First, case and control groups were examined for differences using parametric or non-parametric tests as appropriate for categorical and continuous variables, where *p* ≤ 0.05 was considered statistically significant unless otherwise stated. Next, variables with *p* < 0.1 on bivariate analysis were incorporated in the multivariable analysis. We then used stepwise multiple logistic regression to assess the multivariable relationship between the covariates and the groups. The final multivariable logistic regression model included the parsimonious model with significant covariates (*p* < 0.05) from the stepwise selection process, with PMFQS Ent infection as the dichotomous outcome variable. During the model building stages, we did not find evidence of significant confounding; therefore, the simplest model was chosen based on a relatively small sample size and the effect of variables in the model. All analyses were performed in SAS 9.4 (SAS Institute, Cary, NC, USA).

## Results

### Composition of fluoroquinolone resistance genes in Enterobacterales

We analyzed 170 *bla*-producing Enterobacterales isolates from Hospitals A, B, and C for the presence of FQR genes (**Table 1**). Of 170 Enterobacterales isolates, 85 (50%) were FQR and 85 (50%) were FQS. Of the 85 FQS isolates, 23 (27%) had FQR genes detected (PMFQS cases); and of the 85 FQR isolates, 53 (62%) had FQR genes detected (PMFQR controls).

**Table 1:** Characteristics of PMFQS and PMFQR Enterobacterales.

Variable ^a^	PMFQS Ent	PMFQR Ent
Patient	*n* = 23	*n* = 53
Organism ^b^		
* E. coli*	2 (8.7)	40 (75.5)
* Klebsiella* sp.	13 (56.5)	7 (13.2)
* Proteus* sp.	0 (0)	4 (7.5)
* Enterobacter* sp.	8 (34.8)	2 (3.8)
Source		
* *Urine	12 (52.2)	37 (69.8)
* *Respiratory	8 (34.8)	7 (13.2)
* *Abscess/Wound	0 (0)	3 (5.7)
* *Blood	2 (8.7)	3 (5.7)
* *Peritoneal/Abdomen	1 (4.3)	1 (1.9)
* *Central nervous system	0 (0%)	1 (1.9)
* *Other	0 (0%)	1 (1.9)
Co-antibiotic resistance		
* *Trimethoprim/Sulfamethoxazole	17 (73.9)	38 (71.7)
* *Gentamicin	9 (39.1)	35 (66.0)
* *Amikacin	1 (4.3)	3 (5.7)
* *Carbapenem	1 (4.3)	1 (1.9)
* *MDR (≥3 antibiotic classes)	17 (73.9)	52 (98.1)
*Bla* gene association ^c^		
* *CTX-M-1_group_	8 (34.8)	42 (79.2)
* *CTX-M-9_group_	3 (13.0)	3 (5.7)
* *SHV_ESBL_	10 (43.5)	6 (11.3)
* *TEM_ESBL_	1 (4.3)	0 (0)
* *VEB_ESBL_	0 (0)	1 (1.9)
* *CMY_AmpC_	0 (0)	1 (1.9)
* *ACT/MIR_AmpC_	6 (26.1)	1 (1.9)
* *KPC_CRE_	1 (4.3)	1 (1.9)
* *Multiple *bla* genes	11 (47.8)	7 (13.2)
FQR genes		
* aac(6’)-Ib-cr*	9 (39.1)	44 (83.0)
* oqx A/B*	13 (56.5)	8 (15.1)
* qep A*	0 (0)	7 (13.2)
* qnr A/B/D/S*	7 (30.4)	6 (11.3)
* *Multiple FQR genes	5 (26.1)	7 (13.2)
Chromosomal mutation in QRDR	2 (8.7)	43 (87.8)
Phylogenetic group of *E. coli*	*n* = 2	*n* = 40
* *B2	0 (0)	31 (77.5)
* *D	1 (50)	6 (15.0)
* *A	0 (0)	3 (7.5)
* *B1	1 (50)	0 (0)

a Values represent n (%). Ent, Enterobacterales, PMFQS, plasmid-mediated fluoroquinolone sensitive; PMFQR, plasmid-mediated fluoroquinolone resistant; QRDR, chromosomal mutation in the quinolone resistance-determining region (gyrA and parC); MDR, multidrug-resistant.

b One isolate studied per patient.

c Isolates may harbor one or more bla gene.

Among the subgroups with plasmid-mediated FQR genes detected by PCR, the median age of 23 children with FQS Enterobacterales and the 53 children with FQR Enterobacterales infections was 4.3 and 6.2 years, respectively (*p* = NS). The most common source type was urine, accounting for 52.2% of PMFQS and 69.8% of PMFQR infection sources, followed by the respiratory tract in 34.8% and 13.2% of PMFQS cases and PMFQR controls, respectively. The predominant organisms among PMFQS cases were *Klebsiella* spp. (56.5%) and the most common *bla* genotype was *bla*_SHV ESBL_ in 43.5% of cases, whereas in PMFQR controls, the predominant organisms were *E. coli* (75.5%, *p* < 0.001), and the most common *bla* genotype found associated was *bla*_CTX-M-1-group ESBL_ in 79.2% of controls. The most common quinolone resistance genes among PMFQS Ent and PMFQR Ent isolates were *oqxAB* and *aac(6’)-Ib-cr* in 56.5% and 83.0% of isolates, respectively (*p* < 0.001). Chromosomal resistance mutations in the QRDR region (*gyrA* and *parC*) were found in 8.7% of PMFQS Ent and 87.8% of PMFQR Ent isolates. Multidrug resistance (displayed resistance to three or more antibiotic classes) among PMFQS Ent and PMFQR Ent was common, representing 73.9% and 98.1% of isolates, respectively.

### Whole genome sequencing analysis of PMFQS Enterobacterales Isolates

WGS analysis was performed on 22 of 23 available PMFQS Ent isolates to further analyze genetic determinants of antibiotic resistance (resistome) and the mobile genetic elements associated with propagation of drug resistance. We sought to understand if PMFQS Ent isolates that have non- or low-level gene expression of FQR genes may also display “silent” resistance determinants to other antibiotic classes. Multiple plasmid and strain types were found to be associated with drug resistance among PMFQS Ent isolates (**Table 2**).

**Table 2:** Resistance genes and plasmid types present in PMFQS Enterobacterales isolates.

Organism	MLST	Resistance genes	Plasmid types
*E. cloacae*	114	*aadA2b, aph(3’)-Ib, aph(6)-Id, blaACT-16, blaSHV-12, catA2, dfrA19, fosA, mcr-9, qnrB2, sul1*	IncFII(Yp) IncHI2-IncHI2A Col(pHAD28) IncFIB(pHCM2)
*E. cloacae*	114	*aac(6’)-Ib3, aadA2b, aph(3’)-Ib, aph(6)-Id, blaACT-16, blaSHV-12, E, catA2, dfrA19, fosA, mcr-9, sul1*	IncFIB(pHCM2) IncFII(Yp) Col(pHAD28) IncHI2-IncHI2A
*E. cloacae*	114	*aac(6’)-Ib3, aadA2b, aph(3’)-Ib, aph(6)-Id, blaACT-16, blaSHV-12, dfrA19, fosA, E, mcr-9, qnrB2, sul1*	IncHI2-IncHI2A IncFIB(pHCM2)
*E. cloacae*	117	*aac(6’)-Ib3, aadA2b, aph(3’)-Ia, aph(3’)-Ib, aph(6)-Id, blaACT-16, catB3, blaSHV-12, catA2, dfrA19, fosA, mcr-9, qnrB2, sul1, sul2*	ColRNAI IncHI2-IncHI2A
*E. cloacae*	117	*aac(6’)-Ib3, aac(6’)-IIc, aadA2b, aph(3’)-Ia, aph(3’)-Ib, E, aph(6)-Id, blaACT-16, blaSHV-12, catA2, dfrA19, ere(A), fosA, mcr-9, qnrB2, sul1, sul2*	ColRNAI Col(pHAD28) IncHI2-IncHI2A
*E. cloacae*	148	*aac(6’)-IIc, aadA2b, aph(3’)-Ia, aph(3’)-Ib, aph(6)-Id, blaACT-7, blaSHV-12, dfrA19, E, mcr-9, qnrB2, sul1, sul2*	Col(pHAD28) IncHI2-IncHI2A Col440I
*E. cloacae*	171	*aac(6’)-IIc, aadA2b, aph(3’)-Ia, aph(3’)-Ib, aph(6)-Id, blaACT-16, ere(A), blaSHV-12, dfrA19, fosA, mcr-9, qnrB2, E, sul1, sul2*	Col(pHAD28) IncHI2-IncHI2A
*K. pneumoniae*	17	*aac(6’)-Ib3, aadA2b, aph(3’)-Ib, aph(6)-Id, blaSHV-1, blaSHV-12, blaSHV-96, catA2, dfrA19, fosA, mcr-9, oqxA, oqxB, sul1*	IncHI2-IncHI2A IncFIB(K)-IncFII(K) IncR Col440I
*K. pneumoniae*	17	*aac(3’)-IId, aph(3’)-Ib, aph(6)-Id, blaCTX-M-14, blaLAP-2, blaSHV-1, blaTEM-1B, catA2, dfrA1, fosA, oqxA, oqxB, qnrS1, sul1, sul2, tet(A), tet(D)*	Col440I IncFIB(K)-IncFII(pKP91)
*K. pneumoniae*	29	*aac(6’)-33, aac(6’)-Ib3, aadA2, ant(2’’)-Ia, blaKPC-2, blaSHV-1, blaSHV-187, blaTEM-1A, blaTEM-1D, dfrA12, fosA, oqxA, oqxB, sul1, sul2, tet(D)*	IncFIB(K) IncI1-I(Alpha) IncC IncFIB(pHCM2) ColRNAI
*K. pneumoniae*	45	*blaSHV-1, blaSHV-26, fosA, oqxA, oqxB, tet(D)*	Col440I IncFIB(K)
*K. pneumoniae*	37	*aadA1, blaSHV-1, fosA, oqxA, oqxB, sul1*	Col440I IncFIB(K)
*K. pneumoniae*	268	*aac(3’)-IId, aadA5, aph(3’)-Ib, aph(6)-Id, blaCTX-M-14, blaSHV-1, blaTEM-1B, ere(A), dfrA17, erm(B), fosA5, mph(A), oqxA, oqxB, sul1, sul2, E, tet(A)*	IncFIB(K)(pCAV1099-114) IncFIB(AP001918)-IncFII(pRSB107)
*K. oxytoca*	360	*aac(6’)-Ib3, aac(6’)-IIc, aadA2b, aph(3’)-Ia, aph(3’)-Ib, aph(6)-Id, blaOXY-1-7, catA2, dfrA19, mcr-9, qnrB2, sul1, sul2*	IncFIB(K) IncHI2-IncHI2A IncFII(Yp)
*K. pneumoniae*	429	*aadA1, E, blaSHV-1, dfrA14, fosA, fosA7, oqxA, oqxB, sul1*	Col440I-IncHI1B(pNDM-MAR) ColRNAI FII(pBK30683) IncFIB(pNDM-Mar) FIA(pBK30683)
*K. pneumoniae*	307	*aac(6’)-Ib-cr, aph(3’)-Ib, aph(6)-Id, blaCTX-M-15, blaOXA-1, blaSHV-28, blaTEM-1B, dfrA14, E, fosA, oqxA, oqxB, qnrB1, sul2*	IncFIB(K)-IncFII(K)
*K. pneumoniae*	784	*blaSHV-30, blaSHV-1, fosA, oqxA, oqxB*	IncFIB(K)-IncFII(K) IncM1 IncFIB(pNDM-Mar) IncR
*K. pneumoniae*	133	*blaCTX-M-15, blaSHV-75, blaTEM-1B, fosA, oqxA, oqxB*	ColRNAI IncFIB(K)-IncFII(pKP91) IncI1-I(Alpha)
*K. pneumoniae*	11	*ant(2’’)-Ia, blaSHV-1, blaSHV-30, fosA, oqxA, oqxB*	IncFIB(K)-IncFII(K) IncM1
*K. variicola*	641	*aac(6’)-Ib3, aac(6’)-IIc, aadA2b, aph(3’)-Ia, aph(3’)-Ib, aph(6)-Id, blaLEN16, blaSHV-12, catA2, dfrA19, fosA, mcr-9, oqxA, E, oqxB, sul1, sul2, ere(A)*	IncFII(Yp) IncHI2-IncHI2A IncN IncFIA(HI1) IncFIB(K)(pCAV1099-114)
*E. coli*	3	*aadA5, aph(3’)-Ib, aph(6)-Id, blaCTX-M-15, dfrA17, mph(A), qnrB19, sitABCD, sul1, sul2, tet(A)*	IncFII(pCoo) Col156-IncFIA-IncFIB(AP001918)-IncFII(pRSB107) IncX4 Col(pHAD28) IncI2(Delta)
*E. coli*	24	*aac(6’)-Ib-cr, aph(3’)-Ib, aph(6)-Id, catB3, blaCTX-M-15, blaOXA-1, blaTEM-1B, dfrA8, sul2, tet(B)*	IncFII IncX1 IncFIA IncI1-I(Alpha) IncFII(pHN7A8)

**Figure 1:**
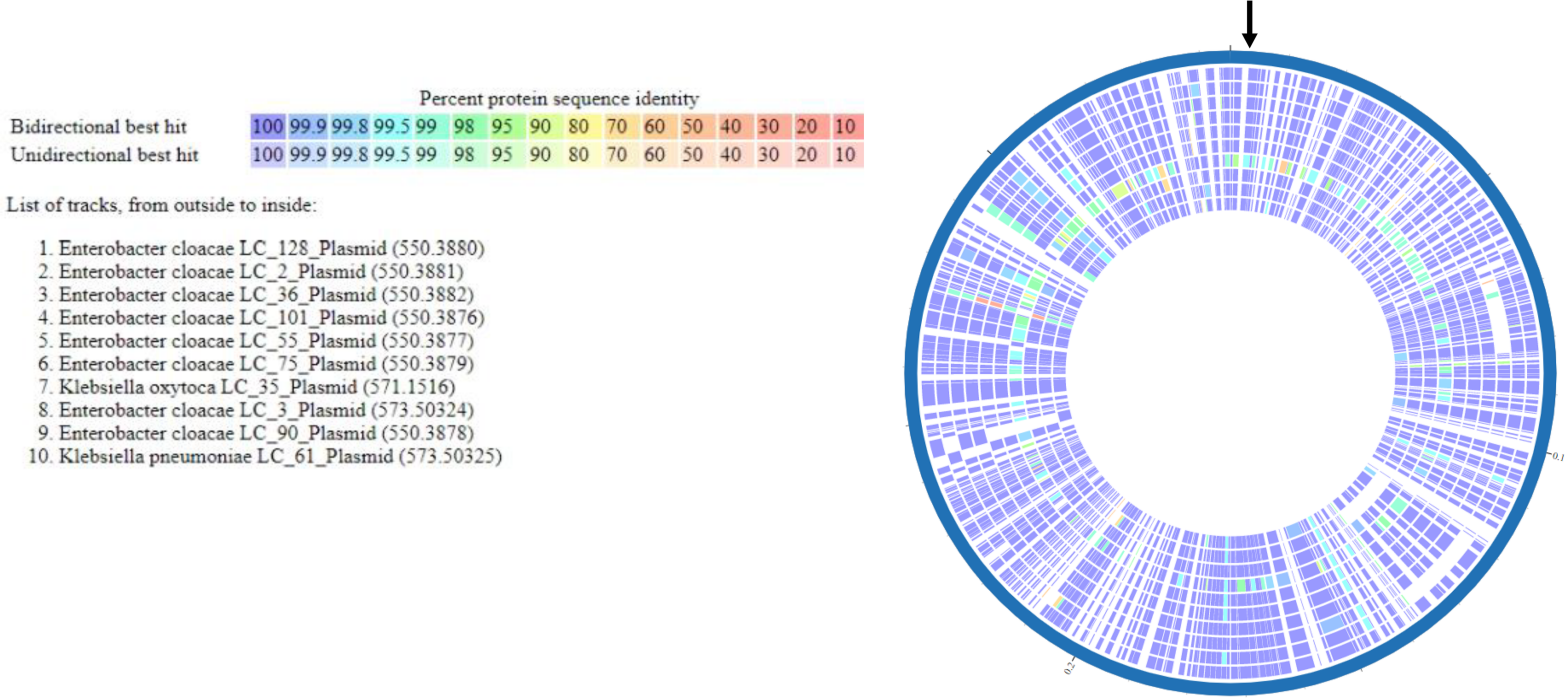
*mcr-9* plasmid proteome comparison among PMFQS Enterobacterales strains generated by the BV-BRC Bacterial Proteome Comparison Service. The location of *mcr-9* is indicated by the black arrow.

**Figure 2:**
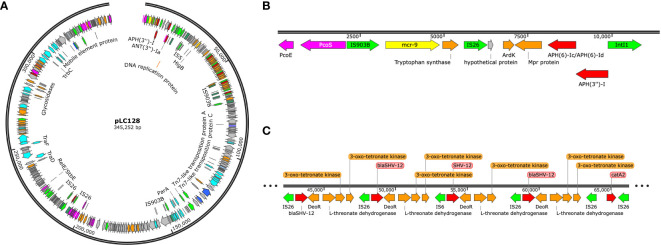
**(A)** *mcr*-9 containing plasmid found in Enterobacter cloacae strain LC128, visualized using Snapgene. **(B)** *mcr*-9 genetic environment. **(C)** *bla*_SHV-12_ gene amplification.

Multi-locus sequence typing of the 22 *E. coli*, *Klebsiella* spp., and *E. cloacae* revealed diverse strain types. The most common plasmid replicons present in strains were IncFI and IncFII. An unexpected finding in the resistome was the presence of *mcr-9* in 10 (43.5%), a gene associated with transferrable polymyxin resistance. The *mcr-9* gene was found in *E. cloacae*, *K. pneumoniae*, and *K. oxytoca* and carried on IncHI2 plasmids. **Figure 1** reveals a close relationship between *mcr-9* harboring plasmids in different strains, based on protein sequence identity, with the *K. oxytoca* strain exhibiting the most significant genetic divergence. **Figure 2** highlights the complexity of the *mcr-9* harboring plasmid and the associated genetic environment.

### Analysis of factors associated with PMFQS Enterobacterales infections in children

The 23 cases of PMFQS Ent infection were compared to 53 controls with PMFQR Ent infections. Significant factors positively associated with PMFQS Ent infection on bivariate analysis included the following: male gender, infection with *Klebsiella* spp. or *Enterobacter* spp., hospital onset infection, location in PICU or NICU settings at the time of diagnosis, comorbid hematologic–oncologic conditions, and having indwelling devices (**Table 3**). Cases with PMFQS Ent infection were less likely than controls to have infection with *E. coli*.

**Table 3:** Bivariate analysis of demographics and factors associated with PMFQS Enterobacterales infection.

Characteristic	PMFQS Ent^c^	PMFQR Ent^d^	*p*-value
Patient	(*N* = 23)	(*N* = 53)	
Median age, years	4.30	6.20	0.43
Hospital			0.89
Male	21 (39.6%)	15 (65.2%)	0.039
Race/ethnicity			0.18
Caucasian	9 (39.1)	14 (26.4)	
African American	5 (21.7)	6 (11.3)	
Hispanic	5 (21.7)	25 (47.2)	
Other	4 (17.4)	8 (15.1)	
Location at diagnosis			0.01
Clinic	3 (13.0)	16 (30.2)	
NICU	3 (13.0)	1 (1.9)	
PICU	12 (52.2)	11 (20.8)	
ED	1 (4.3)	4 (7.5)	
Ward	4 (17.4)	21 (39.6)	
Organism			<0.001
*E. coli*	2 (8.7)	40 (75.5)	
*Klebsiella* sp.	13 (56.5)	7 (13.2)	
*Proteus* sp.	0 (0)	4 (7.5)	
*Enterobacter* sp.	8 (34.8)^a^	2 (3.8)	
Source			0.14
Urine	12 (52.2)	37 (69.8)	
Sputum	8 (34.8)	7 (13.2)	
Other	1 (4.3)	6 (11.3)	
Blood	2 (8.7)	3 (5.7)	
Resistance genes			
*Bla* gene types			0.001
Multiple *bla* genes			0.002
FQR gene types^b^			<0.001
FQR multiple genes			0.36
Recent antibiotics			
Fluoroquinolones^g^	4 (17.4%)	6 (11.3%)	0.48
CTX^f^	11 (47.8%)	15 (28.3%)	0.1
TMP/SMX^h^	8 (34.8%)	8 (15.1%)	0.06
Comorbid conditions			
Cardiovascular	4 (17.4%)	9 (17.0%)	0.97
Gastrointestinal	11 (47.8%)	19 (35.8%)	0.33
Neurological	10 (43.5%)	22 (41.5%)	0.87
Renal	10 (43.5%)	20 (37.7%)	0.64
Pulmonary	10 (43.5%)	17 (32.1%)	0.34
Hematology-oncology	8 (34.8%)	5 (9.4%)	0.01
Endocrine	2 (8.7%)	8 (15.1%)	0.43
Multiple comorbidities	6 (26.1%)	17 (32.1%)	0.6
Invasive devices			
Present	22 (95.7%)	25 (47.2%)	<0.001
Genitourinary	13 (56.5%)	14 (26.4%)	0.013
Central venous catheter	19 (82.6%)	9 (17.0%)	<0.001
Gastrointestinal	13 (56.5%)	13 (24.5%)	0.008
Respiratory	13 (56.5%)	11 (20.8%)	0.002
Recent healthcare			<0.001
None	1 (4.3%)	10 (18.9%)	
Outpatient^e^	5 (21.7%)	31 (58.5%)	
Inpatient	17 (73.9%)	12 (22.6%)	

a Values represent n (%) unless otherwise indicated.

b FQR, fluoroquinolone resistance; PMFQS, plasmid-mediated fluoroquinolone-sensitive infections due to Enterobacterales phenotypically susceptible to fluoroquinolone antibiotics, but found to have fluoroquinolone resistance genes and associated mobile genetic elements present in isolates.

c PMFQR Ent, plasmid-mediated fluoroquinolone-resistant infections due to Enterobacterales phenotypically resistant to fluoroquinolone antibiotics, and found to have fluoroquinolone resistance genes and associated mobile genetic elements present in isolates.

e Outpatient care includes care outside of routine childcare visits and outpatient procedures.

f Includes previous exposure to extended-spectrum cephalosporins (ceftriaxone, ceftazidime, and cefotaxime).

g Includes previous exposure to ciprofloxacin and levofloxacin.

h Includes previous exposure to TMP-SMX, trimethoprim-sulfamethoxazole.

The results of the multivariable regression analysis are shown in **Table 4**. Cases with PMFQS Ent infection were more likely to have hospital-onset infection, defined as infection that began on or later than day 3 of hospitalization (OR 5.7, 95% CI 1.6, 22, *p* = 0.0013). The presence of invasive devices mediated the effects of healthcare setting in the final model. Cases with PMFQS Ent infection were also more likely than controls to have isolates with multiple β-lactamase genes present (OR 3.8, 95% CI, *p* = 0.04), which were often associated with the presence of multiple mobile genetic elements (**Table 2**).

**Table 4:** Multivariable analysis of factors associated with PMFQS Enterobacterales infection.

Associated factor with PMFQS Ent infection	OR	95% CI	*p*-value
Multiple beta-lactamase genes present in isolates	3.8	1.05, 14.5	0.042
Hospital onset infection^a^	5.7	1.6, 22	0.013

^a^Patient was hospitalized for 3 or more days at the time of diagnosis of infection.

## Discussion

In this study, we focused on understanding the genetic basis of FQR in β-lactamase-producing Enterobacterales that were associated with infections in children from multiple centers. We found that among children, there are antibiotic resistance genes on mobile genetic elements that are silently circulating. This non- or low-level expression of resistance is associated with phenotypic susceptibility to antibiotics, yet these resistance genes are transmissible between bacteria, animals, humans, and the environment. Silent dissemination of these plasmids is concerning, especially in a vulnerable population, children. Children are known to be transmitters of many infectious agents, and in the case of MDR Gram-negative bacteria such as the Enterobacterales, children have been found to stay colonized for months to years (Zerr et al., 2014).

Much of the propagation of MDR Enterobacterales is related to the explosion of ESBL-producing bacteria over the past three decades, in particular the ST131 *E. coli* harboring *bla*_CTX-M_ and other high-risk clones containing IncFII plasmids with additional genetic structures such as transposons, integrons, and insertion sequences associated with multiple antibiotic resistance gene cassettes (Lukac et al., 2015). These clonal strains are resistant to multiple antibiotics, including the fluoroquinolones, and this resistance has been found to be both chromosomal- and plasmid-mediated (Canton et al., 2012; Medernach and Logan, 2018).

In adults, the rise of fluoroquinolone-resistant Enterobacterales was first recognized in the 1980s, and the increases were associated with widespread fluoroquinolone overuse. This resistance was mainly due to chromosomally based mechanisms, specifically mutations in *gyrA* and *parC* genes of the QRDR in *E. coli, Klebsiella* spp., and *Enterobacter* spp (Robicsek et al., 2006; Logan et al., 2018; Medernach and Logan, 2018). The most common plasmid-mediated fluoroquinolone resistance (PMFQR) gene in the ST131 *E. coli* is *aac(6’)-Ib-cr*, which encodes an aminoglycoside acetyltransferase, whereby the *cr* mutation additionally confers reduced susceptibility to fluoroquinolones by *N*-acetylation of its piperazinyl amine (Robicsek et al., 2006).

However, we found that among children, the mechanisms of both β-lactam and FQR are diverse, and there is significant horizontal gene transfer occurring among the Enterobacterales genera (Logan et al., 2016; Logan et al., 2019a; Logan et al., 2019b; Logan et al., 2019c; Logan et al., 2020a). Over 25% of isolates phenotypically susceptible to fluoroquinolones harbored an FQR gene. The associated mobile genetic elements carrying these genes were diverse, with most isolates possessing multiple plasmids and other genetic structures such as transposons and insertion sequences. Paradoxically, fluoroquinolone-sensitive Enterobacterales infections were more often hospital onset, while the fluoroquinolone-resistant infections were more often community onset. Additionally, the fluoroquinolone-susceptible Enterobacterales commonly co-harbored multiple β-lactamase and FQR genes and 74% were MDR. Only 9% of fluoroquinolone-susceptible Enterobacterales had chromosomal mutations in the QRDR, i.e., *gyrA* and *parC* (versus 88% of the fluoroquinolone-resistant Enterobacterales). This is not surprising, as typical phenotypic expression of resistance often requires a chromosomal-based mechanism. However, multiple plasmid-mediated genes in Enterobacterales can also be associated with the higher-level expression of resistance, but in our PMFQS cases, 22% of isolates had multiple PMFQR genes that were still found to be susceptible by automated antimicrobial susceptibility systems in the clinical microbiology laboratories, which use CLSI breakpoints (Clinical and Laboratory Standards Institute, 2023).

An unexpected finding was the presence of the mobile colistin resistance (*mcr*) gene, *mcr-9*, in 44% of the PMFQS Enterobacterales infection cases. In November 2015, Liu et al. first described transmissible polymyxin resistance in Enterobacterales associated with the plasmid-mediated colistin resistance gene, *mcr-1*, a member of the phosphoethanolamine transferase enzyme family (Liu et al., 2015). *E. coli* and *K. pneumoniae* that possessed *mcr-1* were found in colonized food animals, contaminated retail meat, and inpatients in five Chinese provinces (Liu et al., 2015; Logan and Weinstein, 2017). The first reported US case of Enterobacterales harboring the *mcr-1* gene was in May of 2016 (Kline et al., 2016), though since this time, others found that *mcr-1* was silently disseminating in Enterobacterales in the US prior to this first report (Castanheira et al., 2016).

Based on data from the National Database of Antibiotic Resistant Organisms (NDARO) (https://www.ncbi.nlm.nih.gov/pathogens/antimicrobialresistance/), *mcr-9* was first detected in a *Salmonella enterica* serovar Schwarzengrund recovered from an avian source in the US in 2014, and the first detection in a human clinical isolate was an *S. enterica* serovar Typhimurium in the US in 2015 (Ling et al., 2020). The majority of the isolates in this current report were infections that occurred in children between 2011 and 2014, suggesting that *mcr-9* was silently disseminating many years in Enterobacterales prior to its first description and that children may have been a factor in the rapid global dissemination of these novel transmissible resistance genes. By 2019, *mcr-9* was the second most widely disseminated among the MCR-family genes (behind *mcr-1*), being identified in isolates recovered from humans, animals, and the environment in 40 countries across six continents (Ling et al., 2020).

While most commonly carried by *Salmonella* species, *mcr-9* has now been detected in several genera of Enterobacterales. The suspected origins of *mcr-9* are livestock and poultry, raising concern that the sources of human acquisition may be linked to community-based environmental influences that include higher exposure risks in certain communities due to certain foods, animals, livestock, water sources, soil, fertilizer, and vegetation (Silbergeld et al., 2008; Ling et al., 2020). Additionally, some of the FQR genes, for example, *oqxA* and *oqxB*, which were the most common FQR genes detected in *Klebsiella* spp. in our study, are multidrug efflux pumps named for their resistance to olaquindox, which is used as a growth promoter on pig farms (Hansen et al., 2005). Furthermore, we conducted prior pilot studies using metagenomic sequencing and culture-based approaches to analyze whether waterways in Chicago used for limited contact recreation (e.g., kayaking, boating, and fishing) were potential reservoirs of MDR Enterobacterales. We found multiple antibiotic resistance genes among Enterobacterales in surface waters, including *mcr-1*, which was detected in three of the four evaluated waterways (Logan et al., 2020b).

In conclusion, we found that children with infections due to β-lactamase-producing Enterobacterales often have other antibiotic resistance genes that may have non- or low-level expression resulting in phenotypic susceptibility to antibiotics, yet these genes are transmissible, and the associated mobile genetic elements and strains are able to silently disseminate. Future studies will assess for the presence of β-lactamase genes, FQR genes, and other antibiotic resistance genes, including MCR-family genes in children and healthy populations, in order to further characterize and validate community sources of MDR Enterobacterales, as well as the risk factors associated with colonization and infection in humans. This will be critical to devise the most effective treatment and prevention strategies for these serious infections in our most vulnerable population, children.

## Data availability statement

The data presented in the study are deposited in the NCBI Genbank repository, under accession number PRJNA989624.

## Ethics statement

The studies involving humans were approved by Ann & Robert H. Lurie Children’s Hospital of Chicago, Rush University Medical Center, Loyola University Medical Center. The studies were conducted in accordance with the local legislation and institutional requirements. The ethics committee/institutional review board waived the requirement of written informed consent for participation from the participants or the participants’ legal guardians/next of kin because this was a retrospective study of salvage isolates obtained for clinical purposes other than study. The study was approved by the institutional review boards of the three participating institutions and the need for informed consent was waived by all institutions.

## Author contributions

All authors listed have made a substantial, direct, and intellectual contribution to the work and approved it for publication.
